# Elemental composition and health risk assessment of PM_10_, PM_2.5_, at different microenvironments: Addis Ababa, Ethiopia

**DOI:** 10.1371/journal.pone.0309995

**Published:** 2024-10-25

**Authors:** Asamene Embiale Taye, Bhagwan Singh Chandravanshi, Feleke Zewge Beshah, Endalkachew Sahle-Demessie

**Affiliations:** 1 Department of Chemistry, College of Natural and Computational Sciences, Woldia University, Woldia, Ethiopia; 2 Department of Chemistry, College of Natural and Computational Sciences, Addis Ababa University, Addis Ababa, Ethiopia; 3 Department of Chemistry, Missouri University of Science and Technology, Rolla, Missouri, United States of America; Chiang Mai University, THAILAND

## Abstract

This study was designed to evaluate the health risks faced by inhabitants living in the slum areas of Addis Ababa, Ethiopia. The levels of PM_2.5_ and PM_10_ and elemental composition of the PM_10_ were measured in indoors (in the kitchen and living room) and outdoors (at the roadside). A total of 75 sampling locations (45 indoor and 30 outdoor) were selected for the study. The levels of PM_2.5_ and PM_10_ were determined using an AROCET531S instrument, while an universal air pump was used for the sampling of PM_10_ for the determination of trace elements by inductively coupled plasma-optical emission spectroscopy (ICP‒OES). The health impacts of PMs on the inhabitants of twelve microenvironments (MEs), where they spend much of their daily time, were estimated. The total amounts of PM_2.5_ and PM_10_, and trace metals in PM_10_ found in the nine or twelve MEs ranged from 10.6–119, 128–185, and 0.007–0.197 μg m^-3^, respectively. According to the United States Environment Protection Agency (USEPA) guidelines, ten of the twelve MEs can cause significant health problems for inhabitants (HI > 1) due to PM_2.5_ and PM_10_. Thus, special attention should be given by stakeholders/inhabitants to minimize the health impacts on long-term exposure. This study assessed the risk of levels of trace elements on the inhabitants who spend most of their daily lives. The study revealed that the lifetime cancer risk values for the individual and cumulative trace elements were within the tolerable range set by the USEPA guidelines.

## Introduction

Air pollution is major concern in the world due to its impact on the longevity of humans and ecosystems [1]. Although the world’s air quality has greatly improved, many regions, especially those in developing countries with dense populations, continue to experience poor air quality [2,3]. Indoor and outdoor air pollution in low- and middle-income countries accounts for 99% and 90%, respectively, of the total deaths worldwide [4]. Chronic and acute exposure to polluted air causes a variety of health complexities in humans, including premature death, neurological disorders, gastrointestinal discomfort, hematological disorders, increased cardiopulmonary morbidity and mortality, dermatitis, and cancer [1,2]. The World Health Organization (WHO) has recently released data showing that 17 people die from air pollution-related causes every minute. The data have also indicated that the life expectancy of populations exposed to high levels of PM_2.5_ decreased the average global life expectancy at birth by approximately 1 year, with decreases of 1.2 to 1.9 years in polluted Asian and African countries [5,6]. Inhabitants of urban areas are much more affected than the rural areas by air pollution due to the expansion of industries, increasing use of biomass as domestic energy, high population density, absence of a good road network, and prevalence of old and poorly maintained vehicles [7–10].

Air pollution in sub-Sahara African (SSA) countries is relatively high due to the use of large amounts of biomass as a fuel source for cooking, limited access to affordable clean fuels, the use of low-efficiency stoves, and the enormous number of old vehicles. The SSA uses approximately 500,000 tons of firewood daily for its daily energy needs [3]. Compared to higher-income homes with effective ventilation systems and a greater likelihood of utilizing high-efficiency stoves, low-income housing is more affected by indoor air pollution [11]. According to a WHO report, the use of inefficient stoves causes the death of 600,000 Africans per year [8,12].

There are variations in the chemical composition of air pollution even within a single nation. This heterogeneity may be caused by varying sociodemographic traits, cultural customs and perceptions, source types and locations, and meteorological circumstances [2,13]. Hence, exposure assessment based on age, sex, activity level, and socioeconomic status is a promising method for remedial action at the individual level. Furthermore, in daily practice, people in the modern world have a much more mobile lifestyle than in recent decades that can be a cause for variation in the contaminant levels of exposure to the inhabitants in multiple locations for different durations. The personal exposure assessment for such people considering a fixed site monitoring method may not be adequately characterized [3,13]. At the same time, the daily activity patterns of individuals have a substantial effect on the total daily intake of pollutants. Thus, spatiotemporal variations in the concentration of contaminants require dynamic cross-ponding measurements of contaminants. Consequently, personal exposure assessments in different microenvironments are a better solution for the determination of pollutants with spatiotemporal variability and for providing more detailed information on individual short-term exposure to indoor and outdoor air pollution [2,3,13]. Therefore, in this study, the individual daily activity patterns in different microenvironments where people spend most of their time are considered in the exposure assessment of the population.

Several studies related to PM_2.5_ and PM_10_ and trace metals bound in the particulate matter in developed and developing countries have been reported in the literature [14–20]. However, there are few studies on air pollution in Ethiopia, and that too without examining the synergetic effects of combined microenvironments on health impact [21–23]. Thus, the synergetic impact of combined MEs, as well as the information on the impacts of respirable air pollutants including PM_2.5_, PM_10_ and heavy metals bound in PM_10_ on inhabitants living in metropolitan city such as Addis Ababa is mandatory. Hence, assessment of exposure to air pollutants using the microenvironmental (ME) modeling approach (average exposure is calculated using time spent and time-averaged concentrations at various places) still needs to be implemented [13]. Moreover, health risk assessments (estimates or predictions) for staying and operating in different combined MEs have yet to be reported in Ethiopia. Therefore, the objectives of this study are as follows: (1) to investigate the levels of PM_2.5_ and PM_10_ and concentrations of trace elements in PM_10_, in the kitchen (during cooking time), living room (feeding time), and roadside (commuting time), (2) to calculate the total dose intake of PM_2.5_, PM_10_, and trace elements in PM_10_ at different combined MEs, and (3) to estimate the potential health risk to inhabitants due to PM_2.5_ and PM_10_, and trace elements in PM_10_ at combined MEs. The results of this study can inform policy options and mitigation strategies to reduce exposure to harmful pollutants effectively and efficiently.

## Materials and methods

### Study region

Addis Ababa, Ethiopia’s capital city, is 2,800 meters above sea level and is situated at 9°1’48"N and 38.74°E latitude and longitude. Addis Ababa city covers 500 km^2^ with 3,000 km of road network, 45.5% of which is asphalt, and 54.5% is a gravel road [22]. The city is the center of many diplomatic and international organizations (the African Union and the World Economic Commission). Human activities, including daily traffic flow, numerous urban constructions, and industrial activities, impact the overall air quality of the city [24,25].

### Microenvironment information

The microenvironments under the study cover the living room (a place where inhabitants eat, study, watch television, discuss and play with their family members), kitchen (an area where inhabitants bake traditional staple food, *Injera*, using different traditional, improved, and clean stoves; cooking traditional sauce called ‘*Wot’* using electricity, kerosene and charcoal of fuels) and roadside (a place where inhabitants are waking, waiting for a taxi and commuting to work during rush hours). These MEs were selected because inhabitants spend most of their daily lives in these places. The pollutant levels in kitchens and roadsides are also expected to be too high. Besides, although the pollutant level in the living room is expected to be low, inhabitants spend more time there than in the kitchen and roadside.

**Fig 1:**
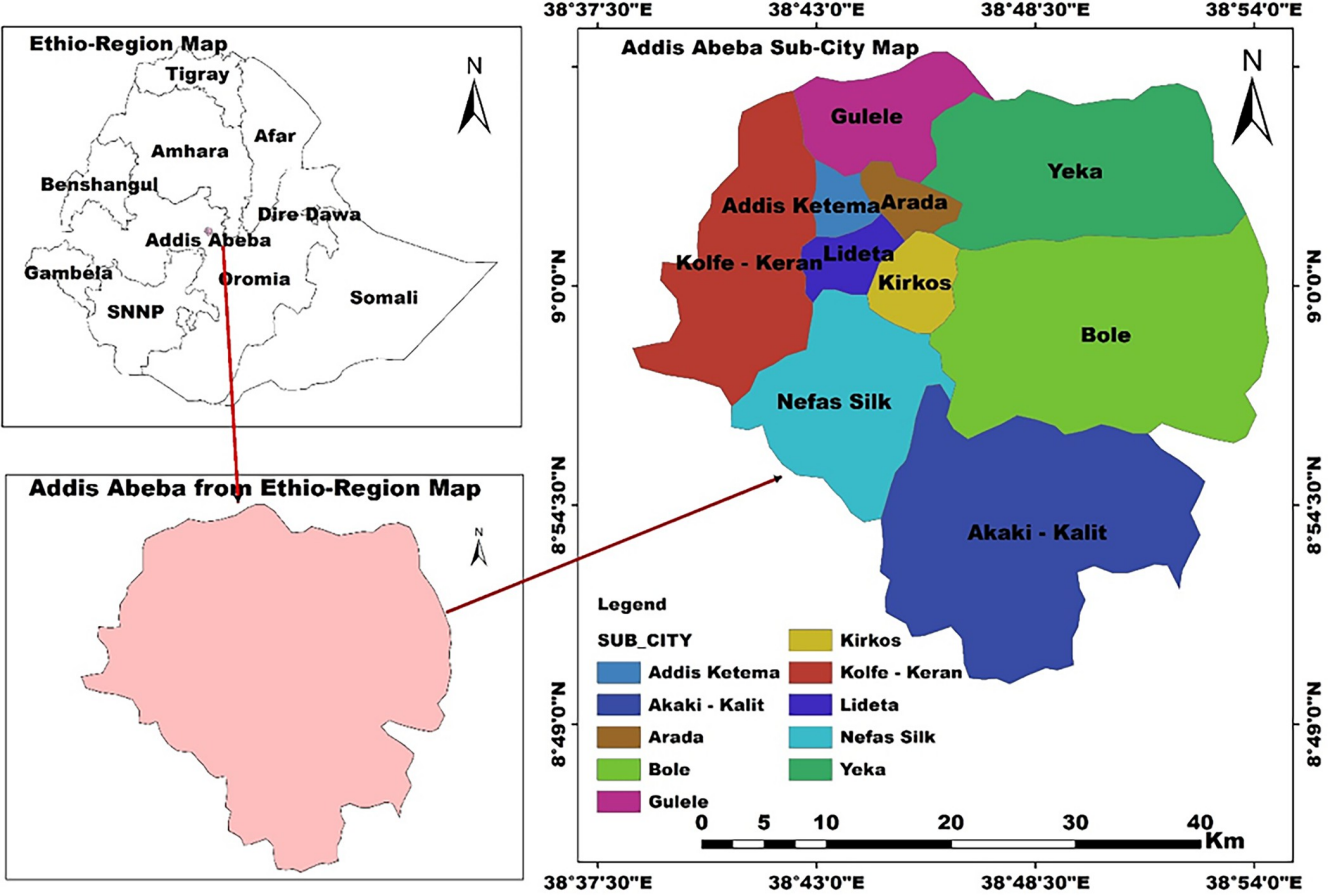
Map showing the sampling area of the ten sub-cities (source: The GADM database).

Furthermore, the type and location of the house selected for the study were mainly based on socioeconomic status, family size, altitude, population density, and the willingness of the owner to allow the researcher. Forty-five households from the three sub-cities (fifteen homes from each) were selected for kitchen and living room MEs. In contrast, thirty sampling locations near the city’s major transportation corridors and roadside locations near the downtown roadside were selected for roadside MEs. A purposive sampling method was employed for selecting houses and sampling locations where the level of pollutants is expected to be high. The determination of levels of PM_2.5_ and PM_10_, and the levels of trace elements in PM_10_ were performed at each ME. Then, the integrated exposure assessment based on the total exposure concentration of the pollutants was calculated using Eq 1. Accordingly, the exposure time and amounts of pollutants are considered [26]. The time spent and level of pollutants could vary by location, affecting the total exposure level of people in these MEs. Consequently, the total levels of exposure are calculated as the product of the sum of time spent by a person in different MEs and the time-averaged air pollution concentrations occurring in those MEs divided by the total time spent in all MEs. The mathematical representation of the concept is given in Eq 1 [13,26]:

$$E_{i}=\frac{1}{T}(\sum_{j=1}^{m}C_{ij}t_{ij})$$ (1)

where *C_ij_* is the concentration of the pollutant measured in the j^th^ ME of the i^th^ individual; *E_i_* is the exposure concentration of the i^th^ individual; T is the total time spent in all MEs; *m* is the number of different MEs; and *t_ij_* is the time spent by the i^th^ individual in the j^th^ ME. Fig 1 displays the sampling region.

### Statistical analysis

The Shapiro‒Wilk test was used to assess the normality of the concentrations of PM_2.5_ and PM_10_, and trace elements in PM_10_. The Kruskal‒Wallis test and analysis of variance (ANOVA) were used to test for significant differences in the concentrations of pollutants across each ME. All the statistical tests were reported by considering a p value of 0.05. PM_2.5_ and PM_10_ were reported as the geometric mean (GOM) and geometric standard deviation (GSD), while the concentration of trace elements in PM_10_ was reported as the arithmetic mean and standard deviation. All the statistical data analyses were performed using SPSS (Statistical Package for Social Sciences), Microsoft Excel 2016, and Microcal TM Origin version 8.

### Measurement of PM_2.5_, PM_10_ and sampling of trace elements in PM_10_

The levels of PM_2.5_ and PM_10_ were determined using AROCET531S (Met One Instrument., OR 07526, USA) within 2 min intervals in each ME. Before entering the field, the instrument was calibrated based on the manufacturer’s operating procedures. The equipment placement was different in each ME’s depending on the inhabitants’ breathing zone. It was 1.5 meters above the ground and 1 m away from the stove during baking of *Injera*, 1 m above the ground, and 1 m away from the stove/seat during cooking of the *Wot*. Similar arrangement was also made in the living room.

An universal air pump (SKC 224-PCTX4 Model, SKC Ltd, U.K.) integrated with a glass microfiber filter (Whatman®, GE Healthcare U.K. Limited, Amersham Place, U.K.) inside was used for sampling PM_10_ for determination of trace elements by ICP‒OES. The sampling device was positioned 1.5 meters above the ground for kitchen (when baking *Injera*) and roadside MEs, which is the average breathing zone for inhabitants. The kitchen (during cooking) and living room MEs were raised to a height of 1 m. The manufacturer’s manual was used to calibrate the instrument’s flow rate [27]. To eliminate the humidity and VOCs on the filters, the filter paper was dried in an oven at 150°C for two hours before sampling. The PM_10_-loaded filters were folded and raped with aluminum foil after the sampling process was complete before being brought back to the laboratory. Finally, to extract the elemental makeup of PM_10_, the wet digestion method developed by the US EPA [28] was used. The literature provides the specifics of the process [27].

### Method validation

The precision and accuracy of the method ensure the data quality. Thus, the instruments were calibrated, standard deviations were evaluated, and recovery tests were performed. A series of working standard solutions, 0, 0.5, 1, 2, 3, 4, and 5 μg mL^-1^ for Ni, Pb, Fe, Cr, Co Cu, Mn, As, Cd, and Sn and 0, 1, 2, 3, 6, 8, and 10 μg mL^-1^ for B, were prepared from a stock solution of 100 mg L^-1^. The linearity (r^2^) of the calibration curves ranged from 0.990 to 0.999, which are acceptable. The method detection limit was calculated using the standard deviation of seven replicate measurements multiplied by three (3 s), which ranged from 0.0001 to 0.003 μg m^-3^. The percent recovery, determined using the spiking method, was in the range of 92 to 110%, which are within the acceptable range. The details of the calibration curve equations and correlation coefficients for each are shown in S1 Table.

### Inhabitants’ health risk assessment of PM_2.5_ and PM_10_

The health risk assessment of inhabitants due to PM_2.5_ and PM_10_ was performed by considering a method established by the U.S. EPA using the microenvironment (ME) modeling method [29]. Thus, MEs were classified based on type, frequency, and time spent for each activity. The most common activities performed by a particular adult are cooking *Wot* (two times per day), baking *Injera* (two times per week), seating in the living room (every day) and commuting to work or another purpose (every day). Consequently, twelve MEs were evaluated (nine possible MEs by considering all activities and three MEs by excluding baking *Injera* from all) to estimate the health risk of exposed inhabitants. The details of each ME and the parameters used in the risk assessment are given in Table 1. The hazard quotient (HQ) and hazard index (HI) values were used for estimating inhabitants’ cancer and noncancer risk. The average daily intake (ADD) in μg kg^-1^ day^-1^ of PM (PM_2.5_ and PM_10_) used for calculating HQ was calculated via Eqs 2 and 3. The HQ and HI values were obtained using Eqs 4 and 9, respectively.

$$ADD=\frac{C\;x\;\mathrm{IR}\;x\;\mathrm{ED}}{\mathrm{AT}\;x\;\mathrm{BW}}$$ (2)

$$ED=\frac{EF\;x\;TA\;x\;EL\;x\;1\;day}{24\;h}$$ (3)

$$HQ=\frac{ADD}{RfD}$$ (4)

where ADD is the average daily intake (μg kg^-1^day^-1^); ED is the exposure duration (days); IR is the intake rate (m^3^ day^-1^); C is the concentration of pollutant in the air (μg m^-3^); EF is the time spent in polluted ME (days year^-1^); TA is the exposure time spent for all activities (hours day^-1^); AT represents the averaging time (DE x 365 days year^-1^); EL is the length of the exposure period (years); 1 day per 24 h is used as the conversion factor; BW is the body weight of inhabitants (60.7 kg, which is the average weight of an African adult); and RfD for PM_2.5_ and PM_10_ are 10 μg m^-3^ and 20 μg m^-3^, respectively [12,28,30]. AT is the average time (67 years, which is Ethiopia’s current average life expectancy) [31].

**Table 1:** The time required for all activity (TA), exposure frequency (EF), body weight (BW), exposure longevity (EL), average time (days), intake rate (IR) and exposure duration (ED).

Code of ME	Inhabitants’ activity	TA (h day-^1^)	EF (days year^-1^)	EL (years)	ED (days)	AT (days)	IR (m^3^ day^-1^)	BW (kg)
‘A’	Cooking *Wot* using electricity fuel, baking *Injera* using clean stove, seating in living room for family discussion, and commuting to work in roadside	10.1	104	50.0	2209	18250	20.0	60.7
‘B	Cooking *Wot* using kerosene fuel, baking *Injera* using clean stove, seating in living room for family discussion, and commuting to work in roadside,	10.3	104	50.0	2253	18250	20.0	60.7
‘C’	Cooking *Wot* using charcoal fuel, baking *Injera* using clean stove, seating in living room for family discussion, and commuting to work in roadside	10.5	104	50.0	2297	18250	20.0	60.7
‘D’	Cooking *Wot* using electricity fuel, baking *Injera* using improved stove, seating in living room for family discussion, and commuting to work in roadside	10.0	104	50.0	2188	18250	20.0	60.7
‘E’	Cooking *Wot* using kerosene fuel, baking *Injera* using clean stove, seating in living room for family discussion, and commuting to work in roadside	10.3	104	50.0	2253	18250	20.0	60.7
‘F’	Cooking *Wot* using charcoal fuel, baking *Injera* using improved, seating in living room for family discussion, and commuting to work in roadside,	10.5	104	50.0	2297	18250	20.0	60.7
‘G’	Cooking *Wot* using electricity fuel, baking *Injera* using traditional stove, seating in living room for family discussion, and commuting to work in roadside,	10.0	104	50.0	2188	18250	20.0	60.7
‘H’	Cooking *Wot* using kerosene fuel, baking *Injera* using traditional stove, seating in living room for family discussion, and commuting to work in roadside and	10.3	104	50.0	2253	18250	20.0	60.7
‘I’	Cooking *Wot* using charcoal fuel, baking *Injera* using traditional stove, seating in living room for family discussion, and commuting to work in roadside.	10.5	104	50.0	2297	18250	20.0	60.7
‘AA’	Cooking *Wot* using electricity fuel, seating in living room for family discussion, and commuting to work in roadside	8.85	365	50.0	6730	18250	20.0	60.7
‘BB’	Cooking *Wot* using kerosene fuel, seating in living room for family discussion, and commuting to work in roadside	9.28	365	50.0	7056	18250	20.0	60.7
‘CC’	Cooking *Wot* using charcoal fuel, seating in living room for family discussion, and commuting to work in roadside	9.07	365	50.0	6904	18250	20.0	60.7

Note: TA and EF for each ME were calculated based on the time required for each activity.

### Assessment of the health risk of inhabitants due to trace elements in PM_10_

PM_10_ is a pollutant that significantly impacts human health, causing serious disease and premature death worldwide [32]. The air pollutants in general and the trace elements in PM_10_ can enter the human body through three main exposure routes: inhalation, ingestion, and dermal absorption [33]. The levels of carcinogenic trace elements, including Cd, As, Pb, Cr, and Ni, and noncarcinogenic elements, including Cu, Fe, Zn, and Mn, bound to PM_10_ were investigated in this study [34]. The U.S. EPA’s integrated risk analysis framework, as expressed in Eqs 5–10, has been implemented to estimate the cancer and noncancer risk of inhabitants by elements in PM_10_ via ingestion, inhalation, and dermal contact exposure routes [35].

$$D_{inh}=\frac{C.InhR.ED.EF}{BW.AT}$$ (5)

$$D_{ing}=\frac{C.IngR.ED.EF}{BW.AT}x10^{6}$$ (6)

$$D_{der}=\frac{C.AF.SA.ABS.ED.EF}{BW.AT}x10^{6}$$ (7)

$${HQ}_{inh\;or\;ing\;or\;der}=\frac{D_{inh}\;or\;D_{ing}\;or\;D_{der}}{RfD}$$ (8)

HI=∑HQ (9)

$$LCRorCR=D_{inh}.IUR=D_{ing}.SF=D_{der}.(\frac{SF}{G})$$ (10)

where C refers to the concentration of elements in the air (μg m^-3^ or mg kg^-1^); D_inh_ refers to the daily dose by inhalation (mg kg^-1^ day^-1)^; D_inge_ refers to the daily dose by ingestion (mg kg^-1^ day^-1^); D_der_ is the daily dose by dermal contact (mg kg^-1^ day^-1^); AT refers to the averaging time (years); inR refers to the ingestion rate (mg day^−1^, 100 for adult); InhR refers to the inhalation rate for adult (20 m^3^ day^-1^); EF refers to the exposure frequency (day.year^-1^, given in Table 1 for each ME); ED refers to the exposure duration in years (18250 days, assumed starting at age of 17); BW refers to the body weight (kg, 60.5 which average body weight of adult African, LCR or CR refers to the lifetime cancer risk due to carcinogenic elements; IUR refers to the inhalation unit risk ((μg m^−3^)^−1^); AF refers to the skin adherence factor (mg cm^−2^ day^−1^, 0.07 for adult adult), AT is the averaging time (ED x life expectence (67 years, for non-cancer risk) and ED x 70, years for cancer risk); RfD is the reference dose of each intake path (mg kg^−1^ day^−1^, give in Table 2 for each element); SF refers to the slope factor (mg kg^−1^ d^−1^, given in Table 2); ABS refers to the dermal absorption factor (unitless, 0.001 for Cd, 0.030 for As and 0.010 for other elements), SA refers to the surface area (cm^2^, 2011 for adult adult), and G refers to the gastrointestinal absorption factor, (unitless, give in Table 2). LCR or CR refers to the chance of an individual developing cancer [35]

**Table 2:** The RfD at different exposure routes and the IUR, SF and G values for different elements [35–37].

Parameter	Fe	Cu	Mn	B	Zn	Pb	Cr	Cd	As	Ni
RfD_Inh_		0.040	0.00005		0.040	0.0035	0.0004	0.00001	0.000015	0.00005
RfD_Ing_	0.070	0.040	0.140	0.200	0.300	0.0035	0.0003	0.001	0.015	0.050
RfD_der_		1.00	1.00		1.00	1.00	0.025	0.025	1.00	0.040
IUR						0.00008	0.012	0.0018	0.043	0.0024
SF						0.280	0.50	0.640	1.50	0.084
G						1.00	0.025	0.025	1.00	0.040

***Note*: *RfD***_***Inh***_**, *RfD***_***Ing***_ ***and RfD***_***der***_ ***are the reference doses for inhalation*, *ingestion and dermal contact exposure*, *respectively*.**

## Results and discussion

### Average levels of PM_2.5_ and PM_10_ at individual microenvironments

**Fig 2:**
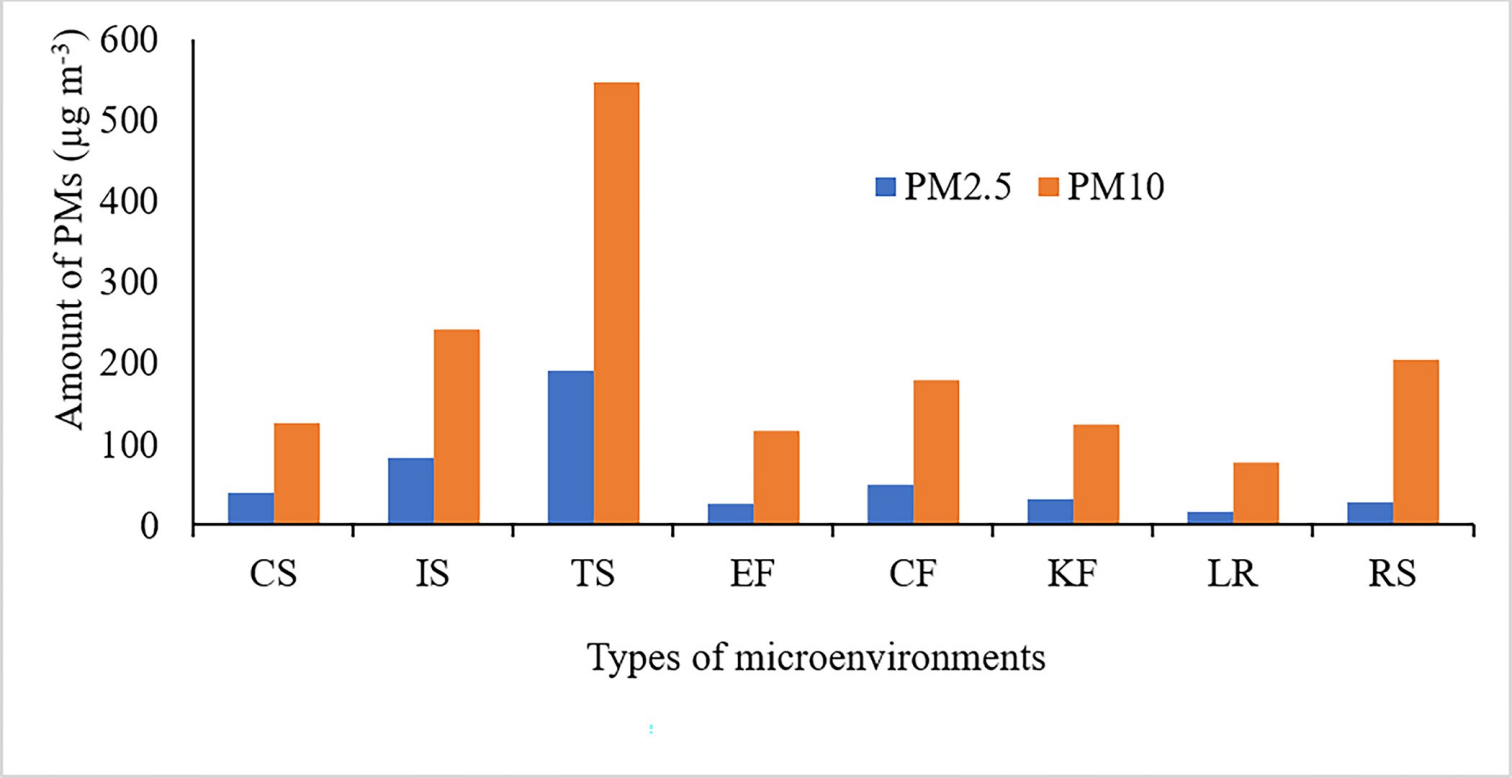
The level of PM_2.5_ and PM_10_ at different microenvironments (CS, IS, TS, EF, CF, KF, LR and RS).

The levels of PM_2.5_ and PM_10_ in different MEs while performing various activities during the study period are given in Fig 2. The microenvironments included LR (living room), EF (kitchen during cooking *Wot* using electricity fuel), KF (kitchen during cooking *Wot* using kerosene fuel), CS (kitchen during cooking *Wot* using charcoal fuel), RS (roadside during commuting), CF (baking *Injera* using a clean stove), IS (baking *Injera* using an improved stove) and TS (baking *Injera* using a traditional stove). The geometric mean of PM_2.5_ was found to range from 17.0 (LR) to 190 (TS) in the following order: LR < EF < RS < KF < CS < VF < IS < TS. The PM_10_ concentration ranged from 77.6% (LR) to 547% (TS) in the following order: LR < EF < KF < CS < CF < RS < IS < TS. The highest amount was recorded at ME using the traditional stove for baking *Injera* due to the smoke coming from incomplete combustion of biomass [38]. Similar results were observed in previously studies that traditional stove has released more pollutants than improved and clean stove [39,40].

### The ADD and total exposure level at combined microenvironments

The ADD, total exposure level and health risk estimation of the inhabitants were carried out based on two scenarios. (1) When an adult spends her daily time baking *Injera*, cooking *Wot*, seating a living room and commuting (A, B, C, D, E, F, G, H and I MEs) and (2) when an adult spends her/his time in the same activity as in scenario 1, excluding baking *Injera* (AA, BB and CC MEs). The daily average time spent by an adult in each MEs was calculated, and the obtained results showed that an adult spent 10.1, 10.3, 10.5, 10.0, 10.3, 10.5, 10.0, 10.3, and 10.5 h in A, B, C, D, E, F, G, H and I MEs, respectively. The results confirmed that 42.0 to 44.0% of adults’ daily life was lost in these MEs. The total exposure levels of PM_2.5_ and PM_10_ in the A, B, C, D, E, F, G, H and I MEs were calculated based on the inhabitants’ exposure times, which ranged from 10.6 to 119 and 128 to 185 μg m^-3^, respectively. The highest value was observed in ME I, possibly due to the use of a large amount of biomass fuel for baking *Injera* and charcoal for cooking *Wot*. Similar result was reported in Kenya by [41], stating that traditional stoves have released large amounts of pollutant.

On the other hand, the total time spent by an adult in the AA, BB and CC MEs was 8.85, 9.28 and 9.08 h, respectively. Thus, the total time spent in the AA, BB and CC MEs accounts for 36.9%, 38.7% and 37.8%, respectively, of the adult’s daily time. The ADDs of PM_2.5_ and PM_10_ ranged from 0.860 to 1.59 and 15.6 to 19.4 μg m^-3^, respectively. The exposure levels of PM_2.5_ and PM_10_ in the AA, BB and CC MEs ranged from 7.08 to 156, with the maximum and minimum values occurring in the CC and AA MEs, respectively, as presented in Table 3. The detailed values for the average daily doses and total exposure levels of PM_2.5_ and PM_10_ at different MEs are given in Table 3.

**Table 3:** The average daily dose (ADD) and total exposure level ([sum (C_ij_xt_ij_)]/T) of PM_2.5_ and PM_10_ at different MEs.

Types of MEs	ADD (μg kg^-1^ day^-1^)		Total exposure (mean ± SD)	
	PM_2.5_	PM_10_	PM_2.5_	PM_10_
A	0.423	5.10	10.6±1.41	128±32.7
B	0.453	5.20	11.1±1.37	128±31.7
C	0.632	5.60	15.3±2.68	135±31.7
D	0.955	5.60	24.2±7.97	142±29.8
E	0.991	5.73	24.4±7.68	141±31.1
F	1.17	6.13	28.2±55.3	148±29.8
G	4.70	6.99	119±7.25	177±33.3
H	4.75	7.13	117±53.9	175±31.9
I	4.93	7.53	119±52.0	185±28.9
AA	0.86	15.6	7.08±1.12	129±42.6
BB	1.59	17.4	12.5±3.68	136±37.3
CC	1.42	19.4	17.6±1.28	156±40.8
RfD_annual_	3.24	6.56	---	----

***Note*: *RfD***_***annual***_ ***is the annual average daily dose*.**

### Health risk assessment of PM_2.5_ and PM_10_ at combined microenvironments

As inhabitants spend a larger proportion of their daily time in the studied MEs, they have high probability of getting non-cancer risk. Table 4 provides a summary of the HQ and HI calculations for the noncarcinogenic hazards of PM_2.5_ and PM_10_ for an adult depending on the total exposure level at each ME. The HQ values is calculated by taking into account of each pollutant’s separate effects. Thus, the HQs of PM_2.5_ and PM_10_ across the MEs (A, B, C, D, E, F, G, H, and I) ranged from 0.130 to 1.52 and from 0.777 to 1.15, respectively. The HQ values of PM_2.5_ and PM_10_ in the G’, H’, and I’ MEs were higher than those in the other units, which indicates that inhabitants in these MEs had a significant noncancer risk. The I and A MEs had the maximum and lowest HQ values, respectively. The combined effects of PM_2.5_ and PM_10_ were evaluated using HI, which was found to range between 0.907 (A) and 2.67 (I) MEs. An adult who spends more time in any of these MEs may be at risk for non-cancer health problems caused by PM_2.5_ and PM_10_, according to the results, which showed that all MEs, with the exception of MEs A and B, have an HI value greater than unit.

**Table 4:** Hazard quotient (HQ) and hazard index (HI) results for PM_2.5_ and PM_10_ at different MEs.

ME	A	B	C	D	E	F	G	H	‘I	AA	BB	CC
HQ for PM_2.5_	0.130	0.140	0.195	0.295	0.306	0.362	1.45	1.47	1.522	0.265	0.490	0.677
HQ for PM_10_	0.777	0.793	0.853	0.853	0.873	0.935	1.07	1.09	1.15	2.38	2.65	2.96
HI (∑HQ)	0.907	0.933	1.05	1.15	1.18	1.30	2.52	2.56	2.67	2.65	3.14	3.64

Furthermore, the noncancer health risk of an adult who did not bake *Injera* was estimated by considering the AA, BB, and CC MEs. The HQs of PM_2.5_ and PM_10_ at the AA, BB, and CC MEs were 0.265–0.677 and 2.38–2.96, respectively. Thus, the HQs of PM_10_ at the AA, BB, and CC MEs were higher than one, and hence inhabitants at these MEs might have a significant health problem. Similarly, the cumulative effects of the PM_2.5_ and PM_10_ concentrations on the AA, BB, and CC MEs ranged from 2.65–3.64, indicating that inhabitants in these MEs might have significant health effects. Therefore, minimize the staying in AA, BB and CC microenvironments is highly recommended to prevent the health impacts.

### The level of trace elements across each microenvironment

**Fig 3:**
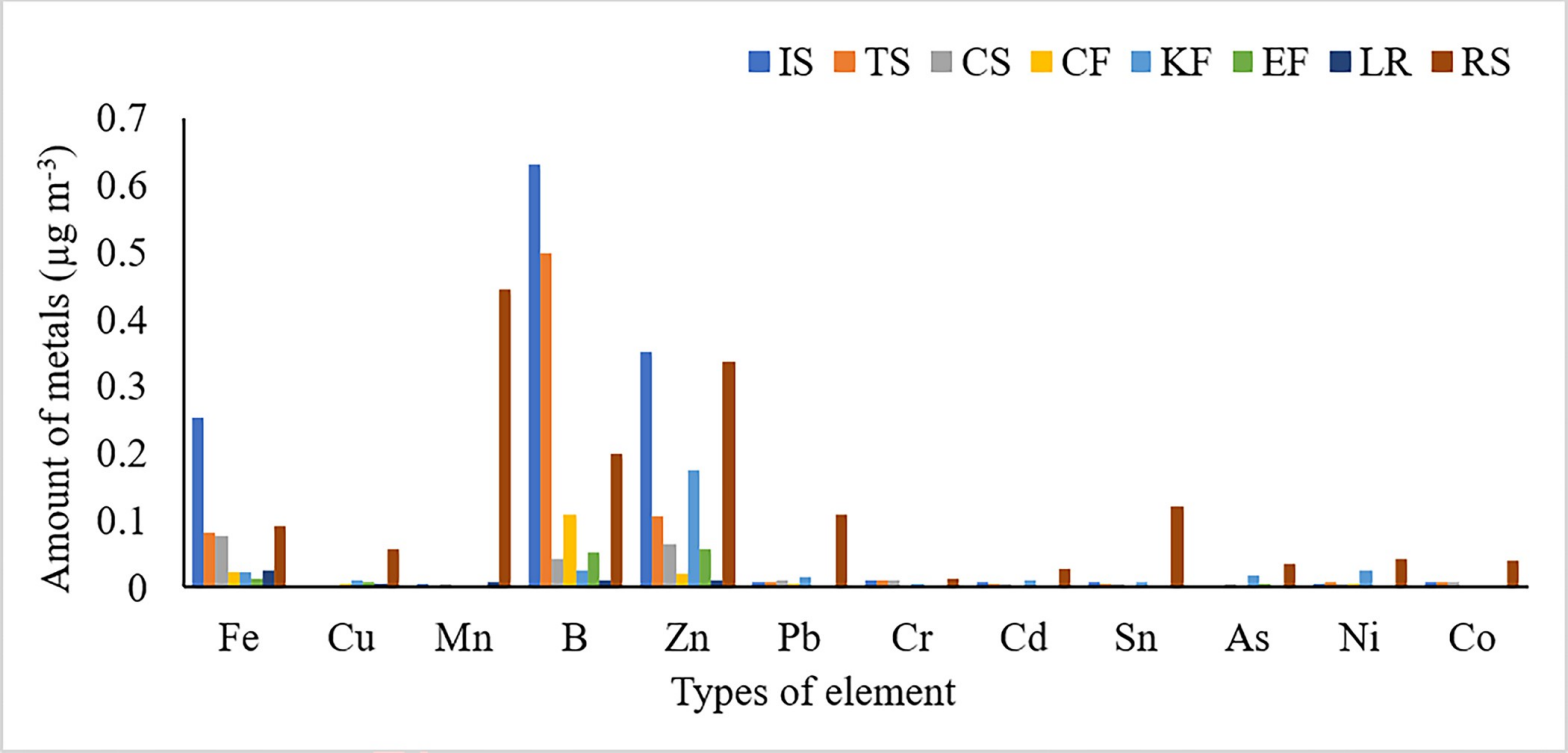
The level of trace elements at different microenvironments (CS, IS, TS, EF, CF, KF, LR and RS).

The corresponding levels of Fe, Cu, Mn, B, Zn, Pb, Cr, Cd, Sn, As, Ni and Co were 0.013 (EF)– 0.254 (IS); BDL (CS, TS and IS)– 0.057 (RS); 0.001 (CF)– 0.444 (RS); 0.01 (LR)– 0.632 (IS); 001 (LR)– 0.351 (IS); 0.001 (LR)– 0.109 (RS); 0.02 (EF)– 0.013 (RS); 0.0007 (EF)– 0.027 (RS); 0.001 (EF)– 0.120 (RS); 0.002 (CF)– 0.036 (RS); 0.003 (LR)– 0.044 (RS); and 0.001 (EF)– 0.04 (RS). The results revealed that the highest levels of most of the trace elements were found in the outdoor microenvironment at the roadside (RS), except for boron and zinc, which were highest in the kitchen microenvironment during baking of *Injera* using an improved stove. This might be due to high traffic congestion, which releases more trace metals through smoke [38]. Similarly, the highest level of B and Zn in improved stove might be due to their micronutrient nature for a plant growth, and that burning of such plant sources resulted for high amount of the metals in particulate matter [42,43]. The general patterns of the investigated elements in different microenvironments are depicted in Fig 3.

### Exposure level of trace elements in PM_10_

The exposure levels of the analyzed elements at the A, B, C, D, E, F, G, H, and I MEs ranged from 0.007 (Cr) to 0.179 (Zn), 0.007 (Cr) to 0.018 (Zn), 0.008 (Cr) to 0.197 (Zn), 0.007 (Cr) to 0.179 (Mn), 0.007 (Cr) to 0.175 (Mn), 0.008 (Cr) to 0.172 (Mn), 0.007 (Cr) to 0.180 (Mn), 0.007 (Cr) to 0.176 (Mn) and 0.008 (Cr) to 0.172 (Mn) μg m^-3^, respectively. The results revealed that the level of Cr was the lowest in all MEs, whereas the levels of Zn in A, B, and C and of Mn in E, F, G, H, and I were the highest. In addition, the total exposure level, regardless of the type of metal, ranged from 0.657 (H) to 0.796 (A) μg m^-3^ in the order of H < I < G < E < F < D < B < C < A. On the other hand, the total exposure levels of the elements in the AA, BB and CC MEs were calculated, and their corresponding values ranged from 0.007 (Cr) to 0.203 (Mn), 0.007 (Cr) to 0.193 (Mn) and 0.007 (Cr) to 0.198 (Mn), respectively. The overall metal exposure levels, regardless of the presence of trace elements, were 0.734, 0.694 and 0.744 μg m^-3^ for the AA, BB, and CC MEs, respectively. The exact values for the total exposure level of each element at each ME are summarized in Table 5.

**Table 5:** The exposure levels of trace elements in combined MEs (mean ± SD μg m^-3^ per microenvironment).

Element	A	B	C	D	E	F
Fe	0.075±0.010	0.073±0.02	0.073±0.016	0.055±0.012	0.053±0.015	0.053±0.014
Cu	0.0242±0.01	0.0243±001	0.0243±0.01	0.024±0.011	0.024±0.086	0.024±0.084
Mn	0.179±0.087	0.176±0.070	0.172±0.080	0.179±0.090	0.175±0.086	0.172±0.084
B	0.170±0.039	0.160±0.040	0.153±0.040	0.155±0.040	0.144±0.04	0.137±0.040
Zn	0.180±0.062	0.182±0.070	0.197±0.060	0.151±0.061	0.154±0.060	0.170±0.058
Pb	0.046±0.021	0.044±0.020	0.045±0.020	0.046±0.021	0.044±0.021	0.045±0.020
Cr	0.007±0.002	0.007±0.002	0.008±0.002	0.007±0.002	0.007±0.002	0.008±0.002
Cd	0.013±0.005	0.012±0.005	0.014±0.005	0.013±0.005	0.012±0.005	0.014±0.005
Sn	0.050±0.023	0.049±0.023	0.049±0.020	0.050±0.024	0.049±0.023	0.049±0.023
As	0.016±0.007	0.016±0.007	0.018±0.007	0.016±0.007	0.016±0.007	0.017±0.006
Ni	0.019±0.008	0.019±0.008	0.023±0.008	0.020±0.008	0.019±0.008	0.022±0.008
Co	0.018±0.008	0.017±0.007	0.017±0.007	0.018±0.008	0.017±0.007	0.017±0.007
	G	H	I	AA	BB	CC
Fe	0.055±0.016	0.052±0.015	0.044±0.015	0.052±0.022	0.048±0.021	0.051±0.021
Cu	0.024±0.010	0.024±0.012	0.033±0.014	0.028±0.014	0.027±0.014	0.028±0.014
Mn	0.180±0.088	0.176±0.086	0.172±0.084	0.203±0.115	0.193±0.110	0.200±0.112
B	0.102±0.0400	0.092±0.040	0.083±0.040	0.110±0.050	0.096±0.040	0.094±0.050
Zn	0.147±0.0640	0.149±0.063	0.162±0.061	0.158±0.086	0.156±0.082	0.182±0.084
Pb	0.046±0.020	0.045±0.020	0.052±0.021	0.051±0.028	0.048±0.027	0.051±0.027
Cr	0.007±0.020	0.007±0.002	0.008±0.002	0.007±0.003	0.007±0.003	0.008±0.003
Cd	0.013±0.005	0.012±0.005	0.014±0.005	0.014±0.006	0.013±0.007	0.015±0.006
Sn	0.050±0.024	0.049±0.024	0.049±0.023	0.056±0.031	0.053±0.030	0.055±0.030
As	0.016±0.007	0.016±0.007	0.018±0.007	0.017±0.009	0.017±0.009	0.020±0.009
Ni	0.020±0.008	0.019±0.008	0.022±0.008	0.021±0.011	0.020±0.010	0.024±0.011
Co	0.017±0.008	0.017±0.008	0.017±0.007	0.019±0.010	0.018±0.010	0.018±0.010

Note: SD is standard deviation.

### Health risk assessment of trace elements

Although baking *Injera* is not a daily routine for inhabitants, it has contributed to the intake of a large amounts of pollutants during the time of baking. As a result, the health risk of the adult was estimated by considering two scenarios: (1) when an adult was baking *Injera* A, B, C, D, E, F, G, H, and I MEs) and (2) when the adult was not baking *Injera* (AA, BB and CC MEs). Thus, inhabitants’ adverse carcinogenic and noncarcinogenic health risks are estimated using three exposure routes (ingestion, inhalation, and dermal contact) at each ME under two scenarios. The study results for scenarios 1 and 2 are shown in Tables 6 and 7, respectively.

**Table 6:** Health risk assessment (for both carcinogenic and noncarcinogenic risks) of heavy metal exposure for inhabitants at different MEs.

A							Total in 3 routes		B						Total in 3 roues	
	Inhalation exposure		Dermal contact		Ingestion exposure		LCR	HI	Inhalation exposure		Dermal contact		Ingestion exposure		LCR*	HI**
Elements	LCR	HQ	LCR	HQ	LCR	HQ			LCR	HQ	LCR	HQ	LCR	HQ		
Fe						9.8E-05		9.8E-05						9.8E-05		9.8E-05
Cu		0.0001		3.1E-07		0.0006		0.0007		0.0001		3.2E-07		0.0006		0.0007
Mn		0.6515		2.3E-06		0.0012		0.6527		0.6625		2.3E-06		0.0012		0.664
B						0.0008		0.0008						0.0008		0.0008
Zn		0.0001		2.3E-06		0.0005		0.0007		0.0001		2.4E-06		0.0006		0.0007
Pb	5.1E-07	0.0024	1.3E-07	5.7E-07	8.3E-06	0.0119	8.9E-06	0.0143	5.2E-07	0.0024	1.3E-07	5.9E-07	8.4E-06	0.012	9.0E-06	0.0144
Cr	1.3E-05	0.0136	1.5E-06	9.6E-07	2.4E-06	0.0023	1.6E-05	0.0159	1.3E-05	0.0137	1.5E-06	9.7E-07	2.4E-06	0.0023	1.7E-05	0.016
Cd	3.2E-06	0.2345	3.3E-07	6.6E-07	5.4E-06	0.0117	8.9E-06	0.2462	3.2E-06	0.2332	3.2E-07	6.6E-07	5.3E-06	0.0117	8.9E-06	0.245
As	9.4E-06	0.1899	6.9E-07	6.0E-07	1.5E-05	0.0475	2.5E-05	0.2374	9.8E-06	0.1976	7.2E-07	6.3E-07	1.6E-05	0.0494	2.6E-05	0.2471
Ni	6.5E-07	0.0708	4.0E-07	6.2E-06	1.1E-06	0.0004	2.1E-06	0.0711	6.6E-07	0.0713	4.1E-07	6.3E-06	1.1E-06	0.0004	2.1E-06	0.0717
HI*	***2*.*6E-05***	1.1629	***3*.*0E-06***	1.4E-05	***3*.*5E-05***	0.0769	6.2E-05^a^	1.2398 ^b^	***2*.*7E-05***	1.1809	***3*.*0E-06***	1.4E-05	***3*.*3E-05***	0.0789	6.3E-05 ^a^	1.2598 ^b^
C									D							
Fe						9.8E-05		9.8E-05						7.2E-05	0	7.2E-05
Cu		0.0001		3.2E-07		0.0006		0.0005721		0.0001		3.1E-07		0.0006	0	0.0007
Mn		0.6497		2.3E-06		0.0012		0.0011601		0.6575		2.3E-06		0.0012	0	0.6587
B						0.0007		0.0007208						0.0007	0	0.0008
Zn		0.0001		2.6E-06		0.0006		0.0006196		9.2E-05		2.1E-06		0.0005	0	0.0006
Pb	5.3E-07	0.0024	1.3E-07	6.0E-07	8.6E-06	0.0122	9.2E-06	0.0122529	5.2E-07	0.0024	1.2E-07	5.9E-07	8.4E-06	0.012	9.0E-06	0.0144
Cr	1.3E-05	0.0145	1.6E-06	1.0E-06	2.6E-06	0.0024	1.8E-05	0.0024377	1.3E-05	0.0136	1.5E-06	9.6E-07	2.4E-06	0.0023	1.6E-05	0.0158
Cd	3.7E-06	0.2657	3.7E-07	7.5E-07	6.1E-06	0.0133	1.0E-05	0.013295	3.2E-06	0.2294	3.2E-07	6.4E-07	5.2E-06	0.0115	8.7E-06	0.2408
As	1.1E-05	0.2213	8.1E-07	7.0E-07	1.8E-05	0.0553	2.9E-05	0.0553473	9.4E-06	0.1898	6.9E-07	6.0E-07	1.5E-05	0.0474	2.5E-05	0.2372
Ni	7.5E-07	0.0818	4.7E-07	7.2E-06	1.2E-06	0.0004	2.4E-06	0.0004116	6.7E-07	0.0726	4.1E-07	6.4E-06	1.1E-06	0.0004	2.2E-06	0.073
HI*	***2*.*9E-05***	1.2366	***3*.*3E-06***	1.6E-05	***3*.*6E-05***	0.0868	6.9E-05 ^a^	0.0869 ^b^	***2*.*6E-05***	1.1654	***3*.*0E-06***	1.4E-05	***3*.*2E-05***	0.0765017	6.2E-05 ^a^	1.2419 ^b^
E							Total in 3 routes		F						Total in 3 routes	
	Inhalation exposure		Dermal contact		Ingestion exposure		LCR	HI	Inhalation exposure		Dermal contact		Ingestion exposure		LCR*	HI**
Elements	LCR	HQ	LCR	HQ	LCR	HQ			LCR	HQ	LCR	HQ	LCR	HQ		
Fe						7.1E-05		7.1E-05						7.2E-05		7.2E-05
Cu		0.0001		3.2E-07		0.0006		0.0007		0.0001		3.2E-07		0.0006		0.0007
Mn		0.6652		2.3E-06		0.0012		0.6663		0.6533		2.3E-06		0.0012		0.6545
B						0.0007		0.0007						0.0007		0.0007
Zn		9.7E-05		2.0E-06		0.0005		0.0006		0.0001		2.2E-06		0.0005		0.0006
Pb	5.2E-07	0.0024	1.3E-07	5.9E-07	8.4E-06	0.0120	9.0E-06	0.0144	5.3E-07	0.0024	1.3E-07	6.1E-07	8.6E-06	0.0123	9.3E-06	0.0147
Cr	1.2E-05	0.0136	1.5E-06	9.6E-07	2.4E-06	0.0023	1.6E-05	0.0159	1.3E-05	0.0144	1.6E-06	1.0E-06	2.6E-06	0.0024	1.8E-05	0.0168
Cd	3.1E-06	0.2268	3.1E-07	6.4E-07	5.2E-06	0.0113	8.6E-06	0.2381	3.6E-06	0.260	3.6E-07	7.3E-07	5.9E-06	0.0130	9.9E-06	0.273
As	9.8E-06	0.1966	7.1E-07	6.2E-07	1.6E-05	0.0491	2.6E-05	0.2457	1.1E-05	0.2206	8.1E-07	7.1E-07	1.8E-05	0.0552	3.0E-05	0.2759
Ni	6.7E-07	0.0728	4.1E-07	6.4E-06	1.1E-06	0.0004	2.2E-06	0.0734	7.7E-07	0.0835	4.7E-07	7.3E-06	1.2E-06	0.0004	2.5E-06	0.0839
HI*	***2*.*7E-05***	1.1774	***3*.*0E-06***	1.4E-05	***3*.*3E-05***	0.0781	6.3E-05 ^a^	1.2556 ^b^	***2*.*9E-05***	1.2346	***3*.*3E-06***	1.5E-05	***3*.*6E-05***	0.0863	6.9E-05 ^a^	1.321 ^b^
G									H							
Fe						7.1E-05		7.1E-05						7.1E-05		7.1E-05
Cu		0.0001		3.1E-07		0.0006		0.0007		0.0001		3.2E-07		0.0006		0.0007
Mn		0.6587		2.3E-06		0.001		0.6599		0.6663		2.3E-06		0.001		0.6675
B						0.0005		0.0005						0.0004		0.0004
Zn		8.9E-05		1.9E-06		0.0004		0.0005		9.4E-05		2.0E-06		0.0005		0.0006
Pb	5.2E-07	0.0024	1.3E-07	5.9E-07	8.5E-06	0.0121	9.1E-06	0.0145	5.2E-07	0.0024	1.3E-07	6.0E-07	8.5E-06	0.0121	9.1E-06	0.0145
Cr	1.3E-05	0.0136	1.5E-06	9.6E-07	2.4E-06	0.0023	1.6E-05	0.0158	1.2E-05	0.0136	1.5E-06	9.6E-07	2.4E-06	0.0023	1.6E-05	0.0159
Cd	3.2E-06	0.2334	3.2E-07	6.6E-07	5.3E-06	0.0117	8.9E-06	0.2451	3.2E-06	0.2381	3.2E-07	6.5E-07	5.3E-06	0.0115	8.8E-06	0.2424
As	9.7E-06	0.1958	7.2E-07	6.2E-07	1.6E-05	0.0490	2.6E-05	0.2448	1.0E-05	0.2027	7.4E-07	6.4E-07	1.6E-05	0.0507	2.7E-05	0.253
Ni	6.6E-07	0.0717	4.0E-07	6.3E-06	1.1E-06	0.0004	2.1E-06	0.0720	6.6E-07	0.0718461	4.0E-07	6.3E-06	1.1E-06	0.0004	2.2E-06	0.0722
HI*	***2*.*7E-05***	1.1757	***3*.*0E-06***	1.3E-05	***3*.*3E-05***	0.0780	6.3E-05 ^a^	1.2538 ^b^	***2*.*7E-05***	1.1878706	***3*.*1E-06***	1.3E-05	***3*.*4E-05***	0.0797	6.4E-05 ^a^	1.2675 ^b^

I							Total in 3 routes	
	Inhalation exposure		Dermal contact		Ingestion exposure		LCR*	HI**
Elements	LCR	HQ	LCR	HQ	LCR	HQ		
Fe						5.97E-05		5.973E-05
Cu		0.000156		4.39E-07		0.000779		0.0009356
Mn		0.65154		2.29E-06		0.001163		0.6527054
B						0.000391		0.000391
Zn		0.000102		2.17E-06		0.000513		0.0006175
Pb	6.0E-07	0.002775	1.5E-07	6.88E-07	9.8E-06	0.013953	1.05E-05	0.016728
Cr	1.4E-05	0.0146	1.6E-06	1.0E-06	2.6E-06	0.002433	1.8E-05	0.0170303
Cd	3.7E-06	0.2699	3.7E-07	7.6E-07	6.2E-06	0.013496	1.0E-05	0.2834123
As	1.1E-05	0.2282	8.3E-07	7.2E-07	1.8E-05	0.057039	3.0E-05	0.2851934
Ni	7.6E-07	0.0821	4.7E-07	7.2E-06	1.2E-06	0.00041	2.5E-06	0.0824858
HI*	***3*.*0E-05***	1.2493	***3*.*4E-06***	1.5E-05	***3*.*8E-05***	0.090237	7.1E-05 ^a^	1.340^b^

*Note*: HI* is the sum of all hazard quotients of all elements in each exposure route; HI** is the sum of all hazard quotients of each element in all exposure routes; LCR* is the sum of the lifetime cancer risk values of each element in all exposure routes; the values in bold and italics indicate the sum of all LCR values of all cancer risk elements in each exposure route; a is the total LCR of all elements by all routes; and ^***b***^ ***is the sum of all trace elements in all routes*.**

**Table 7:** 1Health risk assessment (both carcinogenic and non-carcinogenic risks) form heavy metal exposure of woman via inhalation, ingestion, and dermal exposure pathways at ‘AA’, ‘BB’ and ‘CC’ MEs.

	‘AA’ ME						Total in 3 routes		‘BB’ ME						Total in 3 routes	
	Inhalation route		Dermal route		Ingestion route				Inhalation route		Dermal contact route		Ingestion route			
Elements	LCR	HQ	LCR	HQ	LCR	HQ	LCR*	HI**	LCR	HQ	LCR	HQ	LCR	HQ	LCR*	HI**
Fe						4.5E-05		4.5E-05						4.4E-05		4.4E-05
Cu		8.4E-05		2.4E-07		0.0004		0.0005		8.6E-05		2.4E-07		0.0004		0.0005
Mn		0.4935		1.7E-06		0.0009		0.4944		0.4943		1.7E-06		0.0009		0.4952
B						0.0003		0.0003						0.0003		0.0003
Zn		6.4E-05		1.4E-06		0.0003		0.0004		6.7E-05		1.4E-06		0.0003		0.0004
Pb	3.8E-07	0.0018	9.4E-08	4.4E-07	6.2E-06	0.0088	6.7E-06	0.0106	3.8E-07	0.002	9.3E-08	4.3E-07	6.1E-06	0.0088	6.6E-06	0.0105
Cr	7.9E-06	0.0086	9.3E-07	6.1E-07	1.5E-06	0.0014	1.0E-05	0.010	7.9E-06	0.0085	9.2E-07	6.0E-07	1.5E-06	0.0014	1.0E-05	0.0099
Cd	2.3E-06	0.1641	2.3E-07	4.6E-07	3.8E-06	0.0082	6.3E-06	0.1723	2.2E-06	0.1605	2.2E-07	4.5E-07	3.7E-06	0.0080	6.1E-06	0.1685
As	7.0E-06	0.1411	5.2E-07	4.5E-07	1.1E-05	0.0353	1.9E-05	0.1763	7.2E-06	0.1447	5.3E-07	4.6E-07	1.2E-05	0.0362	1.9E-05	0.1809
Ni	4.8E-07	0.0517	2.9E-07	4.5E-06	7.8E-07	0.0003	1.5E-06	0.0519	4.7E-07	0.0513	2.9E-07	4.5E-06	7.7E-07	0.0003	1.5E-06	0.0515
HI*	***1*.*8E-05***	0.861	***2*.*1E-06***	9.8E-06	***2*.*4E-05***	0.0560	4.4E-05^a^	^b^	***1*.*8E-05***	0.8612	***2*.*1E-06***	9.8E-06	***2*.*4E-05***	0.0566	4.4E-05^a^	0.9179^b^
‘CC’ ME																
Fe						4.5E-05		4.5E-05								
Cu		8.7E-05		2.5E-07		0.0004		0.0005								
Mn		0.4934		1.7E-06		0.0009		0.494								
B						0.0003		0.0003								
Zn		7.5E-05		1.6E-06		0.0004		0.0005								
Pb	3.9E-07	0.0018	9.7E-08	4.5E-07	6.4E-06	0.0091	6.8E-06	0.0109								
Cr	8.6E-06	0.0093	1.0E-06	6.6E-07	1.7E-06	0.0016	1.1E-05	0.0109								
Cd	2.6E-06	0.1884	2.6E-07	5.3E-07	4.3E-06	0.0094	7.2E-06	0.1978								
As	8.2E-06	0.1654	6.0E-07	5.2E-07	1.3E-05	0.0413	2.2E-05	0.2067								
Ni	5.6E-07	0.0603	3.4E-07	5.3E-06	9.0E-07	0.0003	1.8E-06	0.061								
HI*	***2*.*0E-05***	0.9186	***2*.*3E-06***	1.1E-05	***2*.*7E-05***	0.0638	4.9E-05^a^	0.982424^b^								

Note: HI* designates the sum of all hazard quotient of all elements in each exposure routes, HI** designates the sum of all hazard quotient of each element in all exposure routes, LCR* designates the sum of lifetime cancer risk value of each element in in all exposure routes, the values which bold and italic indicates the sum of all LCR values of all cancer risk elements in each exposure routes

^***a***^ ***indicates total LCR of all elements by all routes and***

^***b***^ ***indicates the sum of all trace elements in all routes*.**

### Risk assessment at the A, B, C, D, E, F, G, H, and I microenvironment

The cumulative and individual elemental risk of inhabitants in each ME were assessed based on the method established by the U.S. EPA (i.e., using HQ and HI) [44]. The cancer and noncancer risks to inhabitants from exposure to trace elements were determined for each ME through ingestion, inhalation and dermal contact. The analysis was performed to determine the commutative and individual effects of trace elements using HQ, HI, and LCR values. The cumulative impact is calculated based on each route’s total element intake through all exposure routes (sum of HI of each element) and the total element intake (sum of HI of all elements in a single route). The individual element risk is based on the intake of a single element through each exposure route (HQ_inh_, HQ_ing_ and HQ_der_) and the intake of a single element through the three exposure routes (sum of HQ_inh_, HQ_ing_ and HQ_der_). The obtained results indicated that the adult had a negligible noncarcinogenic risk because the individual element HQ values in both cases for all the elements in all the microenvironments were less than 1. However, the cumulative effect in both cases showed that the HI was greater than one, which indicates that the analyzed elements had a significant health impact on the exposed inhabitants due to their cumulative effect. Different exposure pathways have different contributions to the total exposure level. The dermal contact pathway has the lowest subsidy. Inhalation is the dominant route, followed by ingestion and dermal contact routes, for all MEs. Regarding the individual elements, Mn, Cd and As account for the majority of the commutative effect. The general trends of the HI values followed a decreasing order of I > H > F > C > E > B > G > D > A.

An adult’s cancer risk due to carcinogenic elements was estimated as an individual and cumulative effect in a manner similar to that used for the noncancer risk assessment. LCR values for many individual elements at all MEs through inhalation and ingestion routes were within the tolerable range set by the U.S. EPA (1x10^-6^ to 1x10^-4^). However, except for Cr in dermal contact, the LCR values of all the tested elements are below the limit value (1x10^-6^). The ingestion route is the predominant exposure route, followed by inhalation and dermal contact. The cumulative LCR for all metals was within the tolerable range set by the USEPA. Thus, inhabitants have a low probability of developing cancer in their lifetime. Nevertheless, the LCR values for all the elements are tolerable and below the standard. Furthermore, Cr and Cd contribute more to the LCR. Hence, wearing masks might reduce the intake of PM_10_, minimizing its effect [28].

Similarly, the cancer and noncancer risk of inhabitants who spent time in the AA, BB and CC MEs were also estimated for the A, B, C, D, E, F, G, H and I MEs. The results revealed that both individual elements (a single element in each route and a single element in all routes) and the cumulative effect of trace elements (all elements in a single route and all elements in all routes) in the AA, BB and CC MEs were less than unity, which indicates that a adult in these MEs has limited potential health impacts from noncarcinogenic risk. Mn, Cr, and Cd contributed the most to the total exposure. In addition, although an adult is less likely to have a noncancer risk, inhabitants who spent more time in CC MEs were more likely to have a noncancer risk than inhabitants who spent more on BB or AA MEs. This might be due to charcoal fuel, which contributes to high levels of pollutants. Inhalation is the predominant route of exposure, in contrast to dermal and ingestion. At the same time, many individual elements and their cumulative values of LCR in all routes, except dermal contact, were found to be in the tolerable range (1x10^-6^ to 1x10^-4^) [28]. Cr and As are the two dominant elements for the total exposure routes.

## Conclusion

The total exposure levels of PM_2.5_ and PM_10_ and the elemental composition of PM_10_ at different combined MEs A, B, C, D, E, F, G, H, and I, AA, BB and CC were assessed. The highest exposure levels were observed in I ME (cooking *Wot* using charcoal fuel, baking *Injera* using traditional stove, seating in living room for family discussion, and commuting to work in roadside), that the time spent in this ME should be minimized to reduce the health impacts. The HQs of PM_2.5_ for G, H, and I MEs, and PM_10_ for G, H, I, AA, BB and CC MEs are > 1, indicating that inhabitants at these MEs might have significant non-cancer health problems. The HI values of PM_2.5_ and PM_10_ for all MEs except ‘A’ and B, were >1, revealed that inhabitants at all the MEs, except at the A and B MEs, could have significant health problems due to the synergetic effect of the pollutants. Similarly, the individual metal non-cancer risk assessment showed no significant impact (HQ <1), while the cumulative impact of trace elements showed a significant impact (HI >1) in all MEs. The lifetime cancer risk assessment for carcinogenic elements in all MEs were found within the tolerable range set by U.S. EPA threshold values, which means 1 adult from a million inhabitants can be at risk of developing cancer in her lifetime. Moreover, among the activities, baking *Injera* using traditional stove is the highest pollutant contributor that an immediate alternative solution should be implemented.

Overall, awareness of the public and stakeholders on the health impacts of PM_2.5_ and PM_10_ and elements in PM_10_ at these MEs is highly recommended. The study also suggested that stakeholders look for alternative solutions to reduce air pollution, including promoting the use of clean fuels through minimizing costs, improving of the efficiency of existed stoves, promoting public bus transportation, and reducing taxation of electric cars. Inhabitants also recommended using more efficient and clean stoves, as well as well-ventilated kitchens. Moreover, minimizing the frequency and time spent at MEs with high levels of pollutants (such as G, H, I, AA, BB and CC) is another mechanism for mitigating health problems. Additional studies on the assessment of the toxic organic pollutants in the both indoor and outdoor air and the chemical composition of plant biomass, which is most commonly used as fuel for most Ethiopia, is highly recommended.

## Supporting information

S1 Table The calibration curve equation for the eleven elements in PM_10_. (DOCX)
