# Supplementary material for: Elemental composition and health risk assessment of PM10, PM2.5, at different microenvironments: Addis Ababa, Ethiopia
Source: PLoS One. 2024 Oct 25;19(10):e0309995. doi: 10.1371/journal.pone.0309995 (PMC11508078; doi:10.1371/journal.pone.0309995)
Supplement: S1 Table — (DOCX) [file pone.0309995.s001.docx]

**Elemental Composition and Health Risk Assessment of PM_10_, PM_2.5,_ at Different microenvironments: Addis Ababa, Ethiopia**

**Asamene Embiale Taye^1*^, Bhagwan Singh Chandravanshi^2^, Feleke Zewge Beshah^2^ & Endalkachew Sahle-Demessie^3^**

*^1^Department of Chemistry, College of Natural and Computational Sciences, P. O. Box 400,*

*Woldia University, Ethiopia*

*^2^Department of Chemistry, College of Natural and Computational Sciences, P. O. Box 1176,*

*Addis Ababa University, Ethiopia*

*^3^Department of Chemistry, Missouri University of Science and Technology, Rolla, MO, USA*

**Table S1.** The calibration curve equation for the eleven elements in PM_10_

| Analyte | Calibration equations | Correlation coefficient (r^2^) |
| --- | --- | --- |
| Cu | y = 0.92x + 45 | 0.999 |
| Sn | y = 0.07x + 2.0 | 0.998 |
| Cr | y = 0.46x + 1.0 | 0.991 |
| Pb | y = 0.05x + 63 | 0.990 |
| Cd | y = 1.47x + 63 | 0.997 |
| B | y = 0.08x + 89 | 0.9978 |
| As | y = 0.08x + 14 | 0.998 |
| Zn | y = 0.21x + 10 | 0.992 |
| Ni | y = 0.24x + 14 | 0.995 |
| Mn | y = 1.71x + 78 | 0.999 |
| Fe | y = 0.36x + 10 | 0.999 |
| Co | y = 0.11x + 101 | 0.998 |
